# Cultural perception of triatomine bugs and Chagas disease in Bolivia: a cross-sectional field study

**DOI:** 10.1186/s13071-019-3546-0

**Published:** 2019-06-10

**Authors:** Andrea Salm, Jürg Gertsch

**Affiliations:** Institute of Biochemistry and Molecular Medicine, University of Bern, 3012 Bern, Switzerland

**Keywords:** Chagas disease, Triatominae, *Triatoma infestans*, Knowledge systems, Perception, Vector, *T. cruzi*, Neglected tropical diseases, Bolivia

## Abstract

**Background:**

Chagas disease remains a major public health risk in Bolivia, particularly among rural indigenous communities. Here we studied the cultural perception of the triatomine vectors and Chagas disease among selected rural and urban ethnic groups from different socio-economic and geographical milieus. We focused on the indigenous communities in the Bolivian Chaco where the disease is hyperendemic.

**Methods:**

A cross-sectional study using field observations and structured interviews was carried out among 480 informants in five different regions of Bolivia. Additional semi-structured interviews were conducted. Statistical analyses were performed to determine the correlation of socio-economic variables and indigenous Chagas disease knowledge systems. A total of 170 domestic *Triatoma infestans* vectors were collected and infection with *Trypanosoma cruzi* was analyzed by real-time PCR.

**Results:**

Triatomine bugs were associated with Chagas disease in 70.2% (*n* = 480) of the responses (48.0% Ayoreo, 87.5% Chiquitano, 83.9% Guaraní, 72.2% Quechua, 46.1% La Paz citizens and 67.7% Santa Cruz citizens). Generally, indigenous informants have been educated on the association between triatomine bugs and Chagas disease by institutional anti-Chagas disease campaigns. While communities were largely aware of the vectors as a principal mode of disease transmission, rather unexpectedly, health campaigns had little influence on their prevention practices, apparently due to cultural constraints. Overall, 71.9% of the collected domestic vectors in the Chaco region were infected with *T. cruzi*, matching the high infection rates in the indigenous communities.

**Conclusions:**

Among the Guaraní, Ayoreo and Quechua communities, the groups living in traditional houses have not integrated the scientific knowledge about Chagas disease transmission into their daily hygiene and continue to cohabit with *T. infestans* vectors hyperinfected with *T. cruzi*. An effective translation of Western disease concepts into traditional preventive measures is missing because asymptomatic infections are not generally perceived as threat by the communities. New participatory approaches involving existing ethnomedical knowledge systems could be a successful strategy in the control of *T. cruzi* infection.

## Background

American trypanosomiasis, also known as Chagas disease (CD), is a chronic, systemic, parasitic life-long infection caused by the protozoan *Trypanosoma cruzi* [1]. About 6 to 7 million people are infected worldwide, mostly in Latin America, where the disease is endemic [2]. The parasite *T. cruzi* non-selectively infects mammals and birds and it is mainly transmitted to humans by hematophagous reduviid bugs of the subfamily Triatominae (*Triatoma* spp.) [1, 3]. Transmission occurs subsequent to the insect blood meal when feces deposited by infected bugs come into contact with open wounds or mucous membranes [1, 2, 4]. Although mainly a vector-borne disease, CD also can be acquired by humans through uncontrolled blood transfusions and organ transplantation, congenitally, and through oral contamination [1].

CD remains a major public health concern throughout much of Latin America. It is strongly associated with low socio-economic conditions [5, 6] and results in an economic burden [7]. Bolivia has the highest infection rates in the world and CD occurs in many geographical areas of the country [8–11]. Poor rural dwellings (traditional houses), where vectors progressively adapt and co-exist, are highly associated with parasite infection and CD [12–15]. As a consequence, indigenous communities are more often affected by vector-mediated infection [16–18].

As there is neither a vaccine against infection nor a completely effective treatment for the chronic phase of the disease [19, 20], the Southern Cone Initiative has implemented control strategies that have been focused on the eradication of the vector through insecticide spraying campaigns (mainly pyrethroid insecticides) and screening of blood donors and pregnant women [21, 22]. In 1999, Bolivia launched the Chagas National Programme (Programa Nacional de Chagas) throughout endemic Departments of Bolivia with financial support from the Inter-American Development Bank (Banco Interamericano de Desarrollo, BID) and technical support from the Pan American Health Organization (PAHO). The programme consisted of an initial widespread, massive insecticide spraying campaign in the early 2000’s [23, 24]. In 2006, the elimination of the disease was made a national priority by Bolivia’s CD Law [25]. Besides vector control activities, diagnosis and free treatment were offered in all major cities. However, for the implementation of this initiative, the ethnic socio-cultural aspects of vector control were not taken into account. The knowledge and perception of CD and its vectors by the target population is of great importance for the success and sustainability of CD prevention. Consequently, the WHO has recognized the importance of incorporating social sciences into the study of neglected tropical diseases [26]. Different studies in Latin America have reported on distinct knowledge and belief systems related to CD, showing central factors like the epidemiological severity of the disease, cultural background of the affected ethnic group and presence of vector control and health education activities [27–40]. Despite the CD endemicity in two-thirds of the country, little information is available about the knowledge related to triatomine vectors and CD in Bolivia, particularly in the Bolivian Chaco [41–43]. Infection prevention by vectors has been recognized to demand interdisciplinary, culturally sensitive approaches [26, 44, 45]. Given the fact that CD is a chronic condition, which in only about 30% leads to a lethal cardio-digestive disease and only decades after the first infection [46, 47], the threat of infection is not immediately evident. Here we assessed to which extent selected indigenous ethnic groups in Bolivia associate CD with its triatomine vectors two decades after first vector control intervention.

## Methods

### Study sites and population groups

**Fig. 1:**
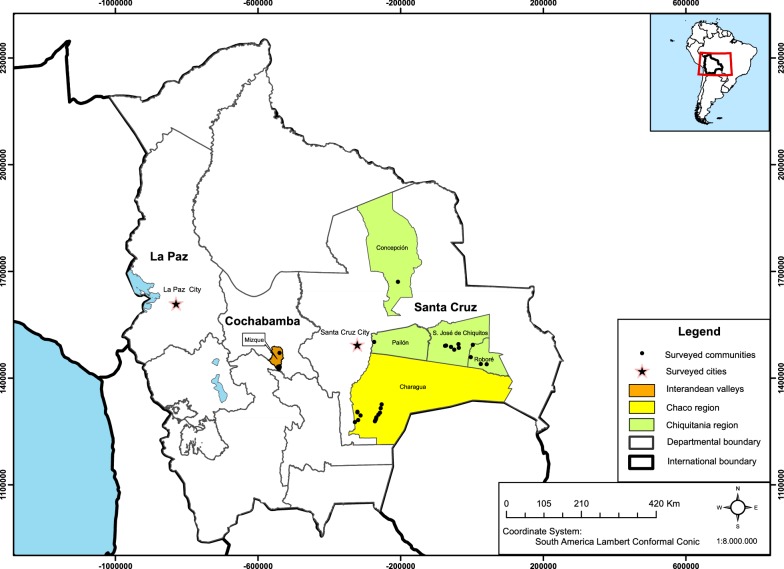
A geographical map showing the study area in Bolivia (8 municipalities within 3 departments) indicating the villages where the survey was carried out

The survey was carried out between June 2014–August 2014 and November 2014–May 2015 in five different regions of Bolivia, in two major settlements (cities of La Paz and Santa Cruz) and in three different geographical rural areas (inter-Andean valleys, Chaco and Chiquitanía region). In 2017 and 2018 we revisited some locations. The communities and respective municipalities where the study was conducted are in the indicated rural zones (Fig. 1). The selection of the study sites was based on the reported incidence CD [9, 15, 48–51], cultural background, accessibility (prior informed consent) and varying degree of acculturation.

In the inter-Andean valleys, the population was native Quechua speaking and largely monolingual, while in the Chaco region the population was primarily of the Native American Guaraní ethnicity. Both are isolated regions due to restrictions in transportation and communication. In the Chaco, also Mennonite colonies are present. Individuals from the Mennonite religious community are of European descent, speak Plautdietsch and have little interaction with local people. Among this group, only unstructured interviews were conducted. In the Chiquitanía region, over 80% of the population belongs to the lowland ethnic group Chiquitano and are native Spanish speakers [52, 53]. Additionally, we included the Ayoreo ethnic group in our survey. Ayoreo people speak Ayoreode dialects of the Zamucoan family [54]. Traditionally nomadic hunter-gatherers, the majority of the communities have been sedentarized and acculturated by missionaries in the mid twentieth century, with the aim to integrate them into Bolivian society. During their process of acculturation, the Ayoreo communities have suffered a traumatic change in their way of live and today are one of the most marginalized ethnic groups in Bolivia. Some striking problems are alcoholism, drug abuse and prostitution, particularly among young Ayoreo people [55]. The survey was carried out in rural Ayoreo communities in the Chiquitanía region and in urban Ayoreo settlements in the city of Santa Cruz de la Sierra. Moreover, the survey was conducted in the cities of Santa Cruz de la Sierra and La Paz. In both cities most of the population is of “mestizo” origin and native Spanish speakers. In the city of La Paz, a large proportion of inhabitants originated from Aymara rural communities.

CD transmission in all studied rural areas was endemic and varied from one region to another, with the highest infection rates reported from the Chaco region and inter-Andean valleys [9, 15, 48–51]. The later region has a history of high rates of human infection with *T. cruzi* [48] and is a reported location of sylvatic *T. infestans* [56–58] and of *T. sordida* [9]. The Gran Chaco has the highest prevalence of CD in the world [50]. Despite the presence of insecticide spraying campaigns in the last 15 years in this region, *T. cruzi* prevalence remains extraordinarily high, with close to universal infection among adults over 30 years-old [15, 49, 59, 60]. In this region *T. infestans*, *T. sordida* and *T. guasayana* have been shown to infest houses [61, 62] and sylvatic populations of these tree species have been described [56, 57, 63–65]. In the Chiquitanía region, *T. sordida* and *T. infestans* have been reported to colonize dwellings [9]. In general, CD is associated with peasant families and rural dwelling infested by triatomine vectors. Nevertheless, migrations to the cities and increase of poverty in the urban areas have also transformed the disease into an increasingly urban health problem [8, 66, 67]. In fact, a study indicates a large prevalence of CD in Santa Cruz city, as an incidence of 23.6% of *T. cruzi* infection was detected in pregnant women [68]. Moreover, the presence of *T. infestans* and *T. sordida* has been reported in the metropolitan area of Santa Cruz city [69, 70]. Owing to the altitude of La Paz city, this region is not an incidence area for triatomine bugs [51].

**Fig. 2:**
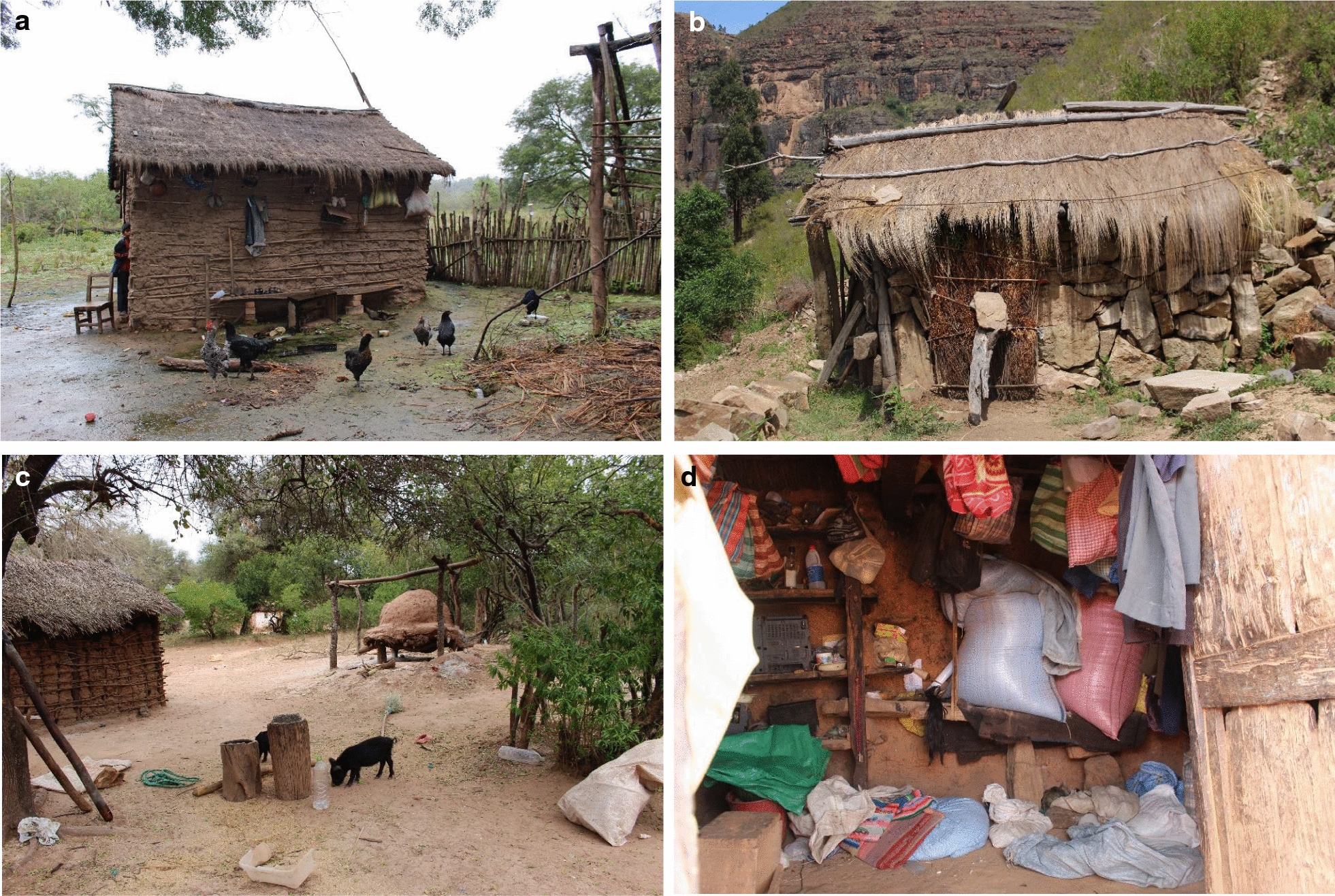
Images of rural dwellings in the study sites. **a** Typical Guaraní house in the Chaco region. **b** Typical Quechua house in the interandean valleys. **c** Peridomicile structure close to a Guaraní house in the Isoso. **d** Interior of a Quechua dwelling

In all three rural areas, dwellings are predominantly constructed of wood and adobe walls, earthen floors and thatched roofs. Therefore, they constitute a suitable habitat for *T. infestans* (Fig. 2). Each household comprises one to four separate structures, usually of one or two rooms. Rooms are dark because of the wall materials and small windows. Animals, which are also hosts to *T. infestans* and other triatomine vectors (primarily *T. sordida* and *T. guasayana* in the Chaco) are raised in close proximity. The peridomestic area comprises several structures such as chicken coops, goat and sheep corrals. In all rural areas, the main economic activity is subsistence agriculture. CD related prevention activities have been carried out during the last decade as part of the Chagas National Programme, mostly as vector control intervention and to a lesser extent (in bigger settlements) serological surveys with pregnant women and school children [21, 24, 51].

In the cities of La Paz and Santa Cruz, public and private hospitals, clinics, medical practitioners and pharmacies provide medical health care. The largest hospitals treat the full range of health issues. In all rural areas, bigger communities have primary health centers adapted to the very basic local needs, which offer children vaccination, assistance of pregnant women and ambulatory care (painkillers, antibiotics, anthelminthic drugs, rehydration salts, etc.). However, most of them are insufficiently equipped. Moreover, large distances and a lack of transport limit accessibility to health services in remote rural areas. In rural areas, respiratory infections, diarrheal diseases and dermatological disorders are the most common medical consultations in primary health centers [51, 71, 72], which indicates the high prevalence of these illnesses. Moreover, after having been integrated to the Bolivian society, the Ayoreo have been exposed to a variety of diseases that were new to them [55]. Two emerging examples are diabetes and AIDS. The first is probably a consequence of the dramatic change of alimentary habits and the second is related to Ayoreo sex workers in the city of Santa Cruz.

### Study design and ethnographical data collection

Ethnographic fieldwork by means of structured and semi-structured interviews provided a basis for the quantitative study. A structured questionnaire was designed to assess the cultural perception of CD and its *Triatoma* spp. vectors among the selected groups of the Bolivian population (Guaraní, Quechua, Ayoreo and Chiquitano indigenous peoples and residents of the cities La Paz and Santa Cruz). It consisted of three major themes: (i) socio-demographic characteristics; (ii) knowledge and perception of triatomine vectors (local names, presence, perceived dangerousness); and (iii) awareness of CD (knowledge, direct experience or indirect through affected relatives, modes of transmission of the disease, sources of information). Questions related to knowledge of triatomine bugs were asked prior to the questions concerning CD in order to avoid bias. A total of 480 people between 10 and 80 years-old were interviewed. Informants were selected consecutively in order of appearance, each representing a different family and according to the prior informed consent, availability and willingness of the informants. A minimum of 50 interviews was carried out for each ethnic group or respectively city. For each visited community, a minimum of 10 representative interviews were conducted with local informants and corroborated by semi-structured interviews (see below). In some very small communities, the minimum number could not be achieved. In urban areas, interviews took place in public places, while in rural communities in the households during the visits. The interviews lasted from 10 to 30 minutes. If possible, interviews were conducted in Spanish, else with the assistance of a local translator. Selected interviews were recorded by video. The informant’s prior informed consent was obtained, and relevant socio-economic data were gathered. In rural areas, semi-structured interviews of approximately 60 to 90 minutes were based on general aspects of the informant’s daily life in relation to and experience with CD, focusing on the knowledge of insect vectors. Interviews related to the ecological knowledge about *Triatoma* spp. were recorded. Additionally, we conducted interviews with local health workers and fumigation technicians. The interviews and observations were registered in field notebooks, photographs or recorded as videos. Upon completion of the structured interviews (see above), the informants were educated about the role of triatomine bugs in CD transmission.

### Data analyses

Structured interview data were collected in Microsoft Office Excel spreadsheets. Percentages were calculated for descriptive statistics. The obtained data were analyzed comparatively. Responses were compared among population groups, which were classified according to people’s ethnicity. Hence, Ayoreo people living in urban settlements were included into the group Ayoreo. In a preliminary analysis, we found no significant differences in the responses of Ayoreo people living in urban or rural settlements. Further, analyses of the influence of socio-demographic and socio-economic factors on informants’ responses were conducted within each population group. We calculated differences by Pearson’s chi-square test of independence or Fisher’s exact test. The connection of triatomine vectors and CD was explored by means of logistic regression analysis (univariate and multivariate) from which odds ratios were computed. A *P*-value of 0.05 was considered as statistically significant. All statistical analyses were conducted using R software version R 3.1.1.

### Determination of infection of *Triatoma* vectors by *T. cruzi*

A total of 170 *T. infestans* bugs were collected during the fieldwork from six rural communities in the Chaco region and from two rural communities in the inter-Andean valleys. Insects were collected from domestic and peridomestic areas (by local residents), transported to the laboratory and stored at − 20 °C before analysis. Dead vectors were dissected with a razor blade for DNA extraction and 20–25 mg of tissue from each insect’s abdomen were transferred in a micro-centrifuge tube with a forceps. In order to avoid cross-contamination, all tools were washed with bleach and rinsed with water after each dissection. DNA extraction followed using the DNeasy kit (Qiagen, Valencia, CA, USA) according to the protocol for animal tissues. The lysis step was conducted over night. DNA yield was determined using a NanoDrop 1000 spectrophotometer (Thermo Scientific, Waltham, MA, USA). Infection with *T. cruzi* was analyzed by real-time PCR. Each PCR reaction was performed in 10 μl containing 5 μl of Fast SYBR® Green Mastermix (Applied Biosystems, Foster City, CA, USA), 0.4 μM *T. cruzi* specific primers TCZ1-F (5′-CGA GCT CTT GCC CAC ACG GGT GCT-3′) and TCZ2 R (5′-CCT CCA AGC AGC GGA TAG TTC AGG-3′), which amplify 188 bp of a repetitive nuclear sequence [73], 30–50 ng DNA and molecular biology grade water. The PCR cycling program consisted of an initial denaturation at 95 °C for 20 s, 40 cycles of 95 °C for 1 s and 60 °C for 20 s. Amplification was followed by a melting program with an initial denaturation at 95 °C for 15 s, cooling to 60 °C for 1 min and then a stepwise temperature increase of 2.63 °C/s from 60 to 95 °C. For each 96-well reaction plate a standard curve was generated from 5 dilutions of positive control. Efficiencies of the amplification were calculated (E = 10^(−1/slope)^). Negative controls consisted of a reaction with *T. cruzi*-specific primers without DNA. Each sample was evaluated in duplicate.

## Results

### General characteristics of informants

Upon prior informed consent, we conducted structured oral interviews based on a questionnaire with 480 informants representing different gender and age groups. Table 1 summarizes the general socio-demographic characteristics of the overall study population.

**Table 1:** Socio-demographic characteristics of the individuals participating in the survey

Socio-demographic characteristics	Ayoreo (*n* = 50)	Chiquitano (*n* = 120)	Guarani (*n* = 87)	Quechua (*n* = 72)	Citizens L.P. (*n* = 89)	Citizens S.C. (*n* = 62)
	*n* (%)	*n* (%)	*n* (%)	*n* (%)	*n* (%)	*n* (%)
Sex						
Male	26 (52.0)	58 (48.3)	26 (29.9)	35 (48.6)	43 (48.3)	33 (53.2)
Female	24 (48.0)	62 (51.7)	61 (70.1)	37 (51.4)	46 (51.7)	29 (46.8)
Age group						
10–20	5 (10.0)	9 (7.5)	7 (8.0)	12 (16.7)	13 (14.6)	11 (17.7)
21–40	18 (36.0)	45 (37.5)	29 (33.3)	34 (47.2)	45 (50.6)	24 (38.7)
41–60	20 (40.0)	35 (29.2)	40 (46.0)	19 (26.4)	21 (23.6)	15 (24.2)
61–80	7 (14.0)	31 (25.8)	11 (12.6)	7 (9.7)	10 (11.2)	12 (19.4)
Educational level						
None	10 (20.0)	0 (0)	5 (5.7)	9 (12.5)	0 (0)	0 (0)
Primary	39 (78.0)	62 (51.7)	74 (85.1)	53 (73.6)	26 (29.2)	13 (21.0)
Secondary	1 (2.0)	48 (40)	7 (8.0)	9 (12.5)	36 (40.4)	30 (48.4)
University	0 (0.0)	10 (8.3)	1 (1.1)	1 (1.4)	27 (30.3)	19 (30.6)

*Abbreviations*: L.P., La Paz; S.C., Santa Cruz

### Knowledge, experience and perception of CD and its vectors

The community knowledge, experience and perception of CD and its vectors were assessed with a questionnaire, accompanied by pictures of two species of triatomine bugs (*T. infestans* and *T. sordida*) in distinct nymphal stages for identification. In cases where the bug was identified, *T. infestans* was the species pointed most frequently. Only in few cases also *T. sordida* was recognized as a different species. A black and less dangerous *vinchuca* was mentioned in the Chaco, possibly referring to *Panstrongylus megistus*. Results of the interviews and differences among the studied population groups are summarized in Table 2.

**Table 2:** Knowledge, perception and awareness of Chagas disease and its triatomine vectors of participants according to their ethnicity

Survey question	Ayoreo	Chiquitano	Guarani	Quechua	La Paz	Santa Cruz	Total	*P*-value
	*n* (%)	*n* (%)	*n* (%)	*n* (%)	*n* (%)	*n* (%)	*n* (%)	*χ*^2^ test
Identification of triatomine bugs								< 0.0001
Yes	46 (92.0)	100 (83.3)	86 (98.9)	65 (90.3)	45 (50.6)	41 (66.1)	383 (79.8)	
No	4 (8.0)	20 (16.7)	1 (1.1)	7 (9.7)	44 (49.4)	21 (33.9)	97 (20.2)	
Have seen triatomine bugs								< 0.0001
Yes	46 (92.0)	98 (81.7)	87 (100.0)	61 (84.7)	31 (34.8)	39 (62.9)	362 (75.4)	
No	4 (8.0)	22 (18.3)	0 (0.0)	11 (15.3)	58 (65.2)	23 (37.1)	118 (24.6)	
“Do you think this bug is dangerous?”								0.0003
Yes	46 (92.0)	114 (95.0)	83 (95.4)	64 (88.9)	70 (78.7)	45 (72.6)	422 (87.9)	
No	2 (4.0)	4 (3.3)	2 (2.3)	4 (5.6)	10 (11.2)	8 (12.9)	30 (6.3)	
Does not know	2 (4.0)	2 (1.7)	2 (2.3)	4 (5.6)	9 (10.1)	9 (14.5)	28 (5.8)	
“Have you heard of Chagas disease?”								< 0.0001
Yes	41 (82.0)	112 (93.3)	83 (95.4)	64 (88.9)	66 (74.2)	59 (95.2)	425 (88.5)	
No	9 (18.0)	8 (6.7)	4 (4.6)	8 (11.1)	23 (25.8)	3 (4.8)	55 (11.5)	
“Do you have a family member who has Chagas disease?”								< 0.0001
Yes	23 (46.0)	43 (35.8)	46 (52.9)	32 (44.4)	12 (13.5)	22 (35.5)	178 (37.1)	
No	24 (48.0)	77 (64.2)	30 (34.5)	27 (37.5)	77 (86.5)	39 (62.9)	274 (57.1)	
Does not know	3 (6.0)	0 (0.0)	11 (12.6)	13 (18.1)	0 (0.0)	1 (1.6)	28 (5.8)	
Association of triatomine bugs and Chagas disease								< 0.0001
Yes	24 (48.0)	105 (87.5)	73 (83.9)	52 (72.2)	41 (46.1)	42 (67.7)	337 (70.2)	
No	26 (52.0)	15 (12.5)	14 (16.1)	20 (27.8)	48 (53.9)	20 (32.3)	143 (29.8)	

#### *Naming triatomines*: general and vernacular nomenclature

*Vinchuca* was the most commonly name used for triatomine bugs and generic for different *Triatoma* species. The term was used in all surveyed regions. Ethnic vernacular names were *timbucu*, used by Guaraní people, *chanyupoay* (*T. infestans*) and *dipeche* (*T. sordida*), used by Ayoreo and *uluchi*, used by Quechua. The last term was mostly referred to nymphal stages of the insect. The Mennonites living in the Chaco area refer to the bug as *steinkwainz*, referring to the Mennonite Low German (Plautdietsch) term for bugs or *bluttier* (blood animal).

#### Triatomine knowledge and experience

**Fig. 3:**
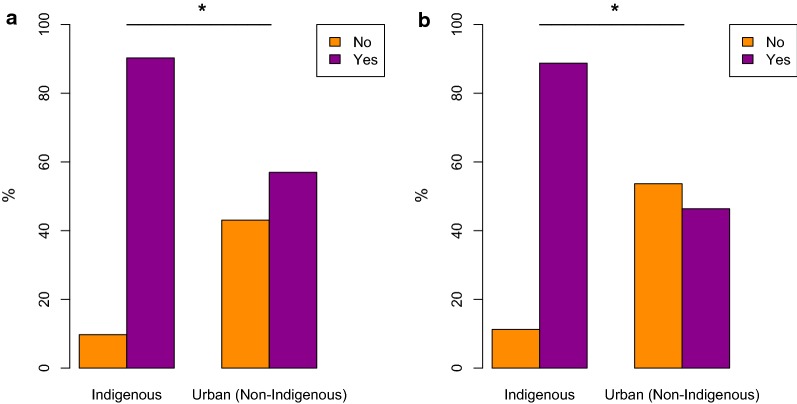
Comparison of knowledge and experience with triatomine bugs between indigenous and urban (non-indigenous) informants. **a** Proportion of informants who correctly identified pictures of triatomine bugs (*n* = 480). **b** Proportion of informants claiming to have seen triatomine bugs (*n* = 480). *Significant association (*P* < 0.05)

Overall, the vector was correctly identified in 79.8% (*n* = 480) of all surveyed subjects (Table 2). However, there were significant differences among the studied groups (*χ*^2^ = 84.4, *P* < 0.0001). Rural indigenous informants from all four studied ethnic groups correctly identified triatomine bugs in over 80% cases, while informants from the cities did this in 50.6% (La Paz) and 66.1% (Santa Cruz). The existence of vectors and the associated experience were examined by asking informants whether they had seen triatomine bugs. As expected, the studied ethnic groups and urban residents of the cities had very distinct direct experiences with the vector and their answers about having ever seen them significantly differed (*χ*^2^ = 126.0, *P* < 0.0001). Expectedly, indigenous people living in rural areas were more knowledgeable of triatomine bugs (*vinchucas*) and claimed to have encountered them more often than people living in the cities (see Table 2, Fig. 3). Informants in the rural areas had a detailed knowledge of *T. infestans* and its habits in general. In all rural regions, some informants mentioned a higher presence of the insect during hot and dry weather conditions, others referred to the blood-feeding behavior, while others mentioned a flying behavior of bugs “coming from the woods to the houses” and some mentioned the night activity of the bugs. In all rural areas, informants affirmed to have seen *vinchucas* inside the houses, by the corrals and in the woods. Many of the affected communities asserted that there was a decrease of triatomine bugs during the last decade and attributed this to the presence of vector control strategies (insecticide spraying). However, vectors were only temporarily eliminated from these communities. In fact, some communities, especially in the Isoso district (Chaco) lived in highly infested dwellings (see below). Generally, reports on number of vectors present in the community varied and could only be assessed by vector sample collection in the houses (see below).

#### Perception of *vinchucas*

In general, *vinchuca* bites were regarded as unpleasant. The *vinchuca* itself was considered dangerous by 87.9% (*P* = 0.0003) of all informants (*n* = 480) when this question was posed. Interestingly, some informants in the Chiquitanía and Chaco region distinguished between red and black *vinchucas* and claimed that only the former were dangerous. The black *vinchucas* might be *Panstrongylus megistus* while the red *vincuchas* were associated to *T. infestans*. In the village of Santiago de Chiquitos (Chiquitanía region) it was often stated that the red *vinchucas* coming from the Chaco region (which arrived together with immigrant workers from that region) are dangerous, while the black *vinchucas* from Santiago are not, since these attack wild animals but no human beings. Informants were asked to identify the reason/s why triatomine bugs are dangerous. Answers are presented in Table 3. CD was spontaneously mentioned by 47.3% (*n* = 480) of the informants (24.0% among Ayoreo, 68.3% among Chiquitano, 58.6% among Guaraní, 44.4% among Quechua, 25.8% among La Paz citizens, 43.5% among Santa Cruz citizens). In general, the relationship of *vinchucas* and a disease was largely identified, even if the disease was not explicitly named. The bite itself as the reason was the answer of 5.8% (*n* = 480) of the interviewees (by 12.0% Ayoreo, 3.3% Chiquitano, 8.0% Guaraní, 4.2% Quechua, 6.7% La Paz citizens and 3.2% Santa Cruz citizens). Some informants (4.2%, *n* = 480) referred to insects carrying a poison, probably due to the acute symptoms after the bite, while others (4.4%, *n* = 480) mentioned the blood-sucking behavior of the bug. Interestingly, among the Mennonites, *vinchucas* were generally not associated with CD but said to suck blood from domestic animals like chicken, which makes them sick.

**Table 3:** Question survey: **“**Why is this bug dangerous?”

	Ayoreo	Chiquitano	Guarani	Quechua	La Paz	Santa Cruz	Total
	% (*n* = 50)	% (*n* = 120)	% (*n* = 87)	% (*n* = 72)	% (*n* = 89)	% (*n* = 62)	% (*n* = 480)
Chagas disease	24.0	68.3	58.6	44.4	25.8	43.5	47.3
Chagas symptoms mentioned	6.0	2.0	4.6	2.8	5.6	1.6	3.8
It has a disease/s	22.0	7.5	13.8	8.3	7.9	6.5	10.2
It makes us feel sick	4.0	0.8	2.3	8.3	na	na	2.3
Lethal disease	4.0	2.5	1.1	5.6	3.4	na	2.7
Bites are a nuisance	12.0	3.3	8.0	4.2	6.7	3.2	5.8
It sucks blood	8.0	0.8	5.7	5.6	1.1	3.2	3.5
Poisonous	4.0	3.3	na	5.6	5.6	9.7	4.4
Appearance	na	1.7	na	na	11.2	3.2	2.9
Every bug is dangerous	na	1.7	na	na	5.6	na	1.5
Other reason	6.0	0.8	1.1	na	4.5	3.2	2.3
Does not know	6.0	3.3	2.3	9.7	14.6	16.1	8.1
Not dangerous	4.0	3.3	2.3	5.6	7.9	9.7	5.2

*Abbreviation*: na, not applicable

Unexpectedly, the large proportion of positive answers about *vinchucas* being dangerous is contradictory to the observed behavior in the field. A natural cohabitation with *vinchucas* in rural Guaraní, Ayoreo and Quechua households and a lack of threat perception of these insects were commonly observed. *Vinchucas* did not awe rural informants and their ubiquitous presence did not seem to disturb them, in contrast to the knowledge about them being dangerous. Many inhabitants were familiar with the bugs and sighting them was a common event. For example, informants were not afraid to touch *vinchucas* with their bare hands. They claimed that *vinchucas* be sometimes disturbing, but they typically coexisted in the house. Consequently, *vinchuca* bites, although unpleasant, were considered normal and frequent. Among the Ayoreo, the vector disease association was significantly lower (Table 3). Moreover, a large proportion of indigenous informants, regardless of the ethnic group, mentioned that they heard that *vinchucas* have a disease or diseases. Some informants were skeptical and did not truly believe that the triatomine bugs were dangerous. As one male Ayoreo informant stated, “*It bit me previously, but I didn’t feel anything of the disease*”. On the contrary, in urban areas (La Paz and Santa Cruz) people clearly perceived *vinchucas* as dangerous and expressed their fear of this bug.

#### Knowledge of CD

The term CD was familiar to the majority of the informants, as 88.5% of the interviewees (*n* = 480) recognized it as a disease. In agreement with the previous questions, many informants indicated *vinchuca* bites as the main mode of transmission. Guaraní and Chiquitano people were in general well aware of CD in their communities and recognized its different clinical manifestations. However, among Quechua and Ayoreo informants, CD and its transmission seemed not always to be clear concepts. Some interviewees claimed to have heard about the illness, although they were not sure about the underlying pathology and symptoms, and transmission mechanisms. Altogether, only 9 informants (1.9%) explicitly stated that the *vinchuca* feces transmit the illness. Transmission through blood or congenitally was mentioned by a small proportion of the interviewees (1.3% and 1.5%).

Overall, a high proportion of informants (37.1%, *n* = 480) claimed either to suffer personally from CD or to have family members or relatives who were affected by the illness (Table 2). Especially, the studied communities in the region Chaco were severely affected. 52.9% of the interviewees had CD history in their families. As one male Guaraní (aged 45) stated, “*here almost everybody has Chagas*”. The majority of the affected people in the Chaco and Chiquitanía region suffered from heart disease, while a large proportion of the affected Quechua population suffered from digestive or mixed alterations. Mostly, people diagnosed with CD learnt about their condition after tests conducted at pregnancy, blood donation and sometimes at serological surveys in schoolchildren or in workers, whose employers required such tests. In some communities some informants remembered having been tested, but stated that they never got the results. In more remote locations, informants were never tested. In some Guaraní and Quechua informants there was a suspicion based on the symptoms.

The informants gave a wide range of sources of information related to CD. In indigenous communities, health facilities and health promoters were the most prominent sources (principal transmitters). For example, a female Guaraní (aged 41) stated, “*sometimes doctors tell us that there is Chagas in Isoso*”. Another female Guaraní (aged 30) remembered, “*about two years ago they came here and taught us, but I have already forgotten*”. Quechua and Guaraní informants mentioned announcements about CD over the radio as an important source of information. Ayoreo people mentioned health education workshops organized by external institutions at community meetings as another sources of information. Other informants in rural areas acquired their knowledge from technicians from spraying campaigns.

### Association between triatomine vectors and CD

The data from the structured interviews were analyzed to assess the perceptual association between *vinchucas* and CD. Responses that mentioned the relationship of triatomine bugs with CD or a chronic condition without naming the disease scored as positive answers. Many informants had heard about the term CD and about its relation to *vinchucas*. If they did not know the health consequences and/or clinical manifestations of the disease, their answers scored negative (no association).

**Fig. 4:**
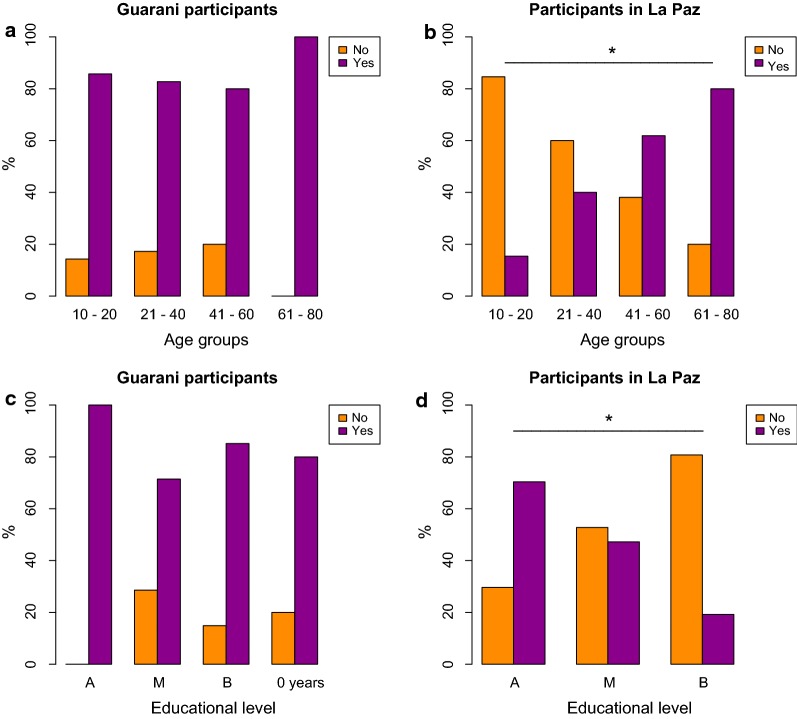
Influence of age and educational level on Guaraní and La Paz informants’ association of triatomine bugs and Chagas disease (CD). **a** Age and **c** educational level had no influence among Guaraní informants (*n* = 87*)* while both variables had an influence among informants in La Paz (*n* = 89), **b** and **d**. *Significant association (*P* < 0.05)

Overall, *vinchucas* have been positively associated with CD in 70.2% cases (*n* = 480). However, there were significant differences (*χ*^2^ = 70.2, *P* < 0.0001) among the population groups, as 48.0% Ayoreo, 87.5% Chiquitano, 83.9% Guaraní, 72.2% Quechua, 46.1% La Paz citizens and 67.7% Santa Cruz citizens could connect *vinchucas*’ bites with a chronic health condition. Further, binomial univariate and multivariate logistic regression analyzes were performed in order to determine which socio-demographic and socio-economic factors influence the association between *vinchucas* and CD within each studied population group (Table 4). Among Guaraní and Quechua informants, gender, age group and educational status were not significant (*P* > 0.05). We detected significant gender differences in the univariate regression analysis among the Ayoreo, Chiquitano and La Paz groups (*P* < 0.05). Within these three groups, males associated *vinchucas* with CD more often than females. After univariate logistic regression analysis, age had a significant influence (*P* < 0.05) on the responses among the Chiquitano informants and residents of La Paz and Santa Cruz. Among the La Paz group, the association between CD and its vector linearly increased with the age of the informants. The educational background clearly had an influence only among informants living in the surveyed urban areas in the univariate analysis (*P* < 0.05). La Paz and Santa Cruz citizens associated *vinchucas* and CD more frequently with increasing educational level. In general, the responses among rural interviewees appeared to be more homogeneous in comparison to urban interviewees (Fig. 4). The results of the multivariate analysis indicated that the variables age and educational level remained significant (*P* < 0.05) among urban informants from La Paz and Santa Cruz (Table 4). The factor gender remained significant in the final regression model among the Ayoreo and Chiquitano informants as only 29.2% of Ayoreo female informants connected triatomine vectors with CD.

**Table 4:** Univariate (unadjusted OR) and multivariate (adjusted OR) logistic regression to identify socio-economic variables for awareness of triatomine bugs as vectors of Chagas disease

Demographic background	% who connected	Unadjusted		Adjusted	
	Bugs with Chagas	OR	95% CI	OR	95% CI
Ayoreo participants					
Age					
≤ 40*	34.8	Reference		Reference	
41–60	65.0	3.48	1.02–12.89	3.84	1.01–16.58
61–80	42.9	1.41	0.23–8.02	1.50	0.15–14.82
		*P* = 0.14		*P* = 0.15	
Gender					
Female	29.2	Reference		Reference	
Male	65.4	4.59	1.44–16.00	4.79	1.40–18.67
		*P* = 0.012		*P* = 0.016	
Education					
No formal education	40.0	Reference		Reference	
Basic or higher	50.0	1.50	0.37–6.64	1.30	0.20–9.44
		*P* = 0.57		*P* = 0.78	
Guaraní participants					
Age					
10–20	85.7	1.12	0.16–22.56	1.51	0.18–34.53
21–40	82.8	0.89	0.27–3.24	0.88	0.24–3.41
> 40^a^	84.3	Reference		Reference	
		*P* = 0.97		*P* = 0.92	
Gender					
Female	83.6	Reference		Reference	
Male	84.6	1.08	0.32–4.27	1.07	0.28–4.68
		*P* = 0.91		*P* = 0.92	
Education					
No formal education	80.0	Reference		Reference	
Basic	85.1	1.43	0.07–10.88	1.39	0.07–10.61
Intermediate and higher^a^	75.0	0.75	0.03–10.78	0.61	0.02–10.13
		*P* = 0.74		*P* = 0.68	
Chiquitano participants					
Age					
10–20	44.4	0.03	0.00–0.22	0.02	0.00–0.16
21–40	88.9	0.27	0.01–1.77	0.22	0.01–1.67
41–60	88.6	0.26	0.01–1.87	0.30	0.01–2.31
61–80	96.8	Reference		Reference	
		*P* = 0.0071		*P* = 0.1	
Gender					
Female	80.6	Reference		Reference	
Male	94.8	4.40	1.31–20.13	5.05	1.25–29.97
		*P* = 0.028		*P* = 0.03954	
Education					
Basic	83.9	Reference		Reference	
Intermediate and higher^a^	91.4	2.04	0.68–6.92	3.64	0.96–16.50
		*P* = 0.22		*P* = 0.069	
Quechua participants					
Age					
10–20	50.0	Reference		Reference	
21–40	70.6	2.4	0.62–9.55	2.76	0.65–12.27
41–60	89.5	8.5	1.51–70.24	10.64	1.73–95.18
61–80	71.4	2.5	0.37–22.74	3.07	0.41–30.58
		*P* = 0.16		*P* = 0.13	
Gender					
Female	64.9	Reference		Reference	
Male	80.0	2.17	0.76–6.60	1.82	0.54–6.36
		*P* = 0.16		*P* = 0.33	
Education					
No formal education	66.7	Reference		Reference	
Basic	69.8	1.16	0.22–4.98	1.19	0.20–6.41
Intermediate or higher	90.0	4.5	0.45–103.84	5.69	0.46–151.45
		*P* = 0.44		*P* = 0.37	
La Paz participants					
Age					
10–20	15.4	0.05	0.00–0.32	0.02	0.00–0.22
21–40	40.0	0.17	0.02–0.76	0.14	0.01–1.00
41–60	61.9	0.41	0.05–2.15	1.65	0.14–19.65
61–80	80.0	Reference		Reference	
		*P* = 0.014		*P* = 0.0043	
Gender					
Female	34.8	Reference		Reference	
Male	58.1	2.60	1.12–6.25	2.51	0.80–8.45
		*P* = 0.029		*P* = 0.12	
Education					
Basic	19.2	0.10	0.03–0.34	0.05	0.01–0.25
Intermediate	47.2	0.38	0.13–1.06	1.62	0.41–6.92
Advanced	70.4	Reference		Reference	
		*P* = 0.002		*P* = 0.0014	
Santa Cruz participants					
Age					
10–20	63.6	0.22	0.04–1.21	0.14	0.01–1.03
21–40	45.8	0.11	0.02–0.40	0.06	0.01–0.30
> 40^a^	88.9	Reference		Reference	
		*P* = 0.0096		*P* = 0.009	
Gender					
Female	62.1	Reference		Reference	
Male	72.7	1.63	0.56–4.86	0.88	0.23–3.25
		*P* = 0.372		*P* = 0.85	
Education					
Basic	38.5	0.17	0.03–0.75	0.09	0.01–0.63
Intermediate	73.3	0.73	0.17–2.79	0.88	0.16–4.70
Advanced	78.9	Reference		Reference	
		*P* = 0.05		*P* = 0.035	

^a^Levels grouped together because of small sample size

*Abbreviations*: OR: odds ratio, 95% CI: 95% confidence interval

### Infection of vectors with *T. cruzi*

**Fig. 5:**
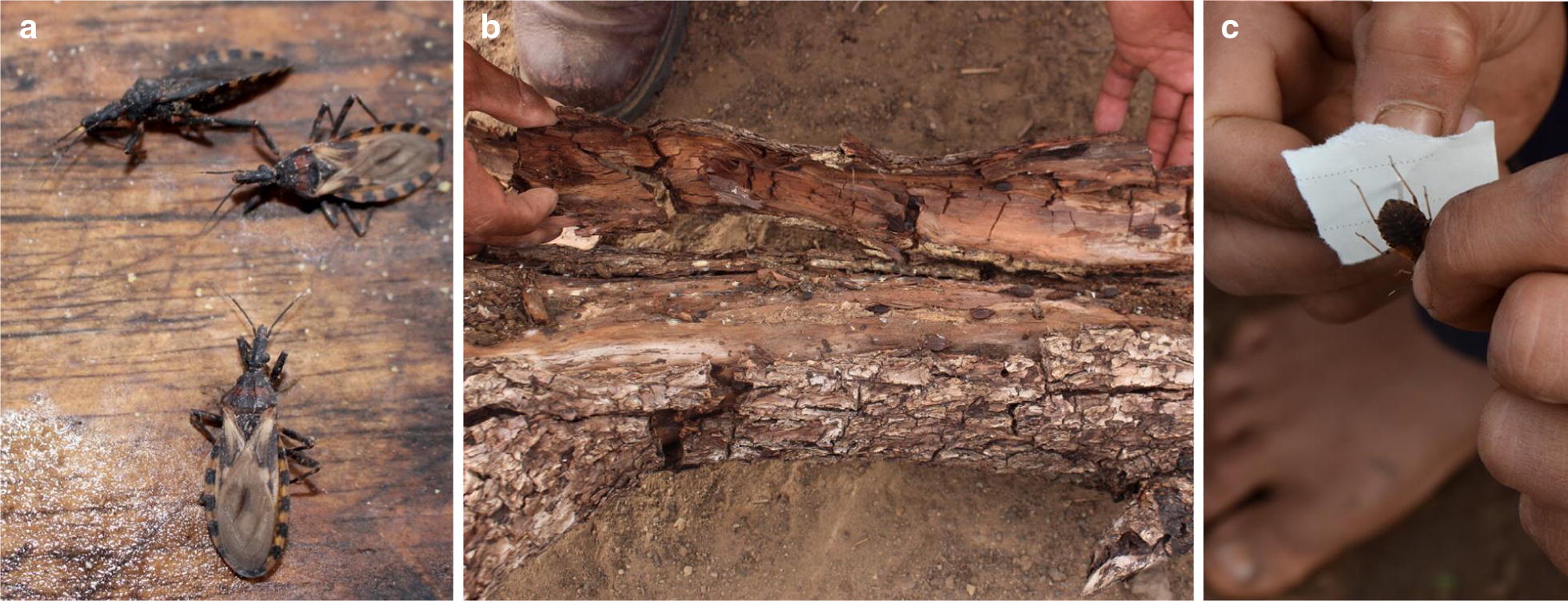
Images of triatomine vectors. **a** *T. infestans* collected in a Guaraní dwelling in the Chaco. **b** Living nymph stage *T. sordidas* found inside tree trunk which was part of a structure of a sheep corral in the peridomestic area (Mizque municipaty). **c** *T. infestans* collected in a Guaraní house for subsequent real-time PCR analysis

**Fig. 6:**
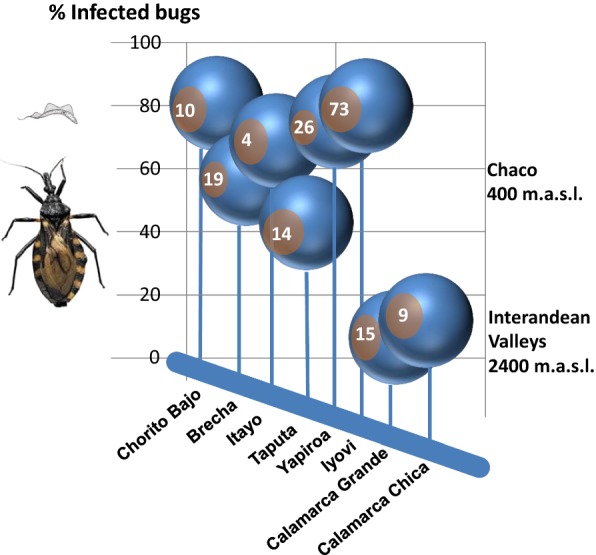
Geographical origin of triatomine bugs and infection rate determined with real-time PCR (% of total). Numbers indicate amount of bugs collected from 2–5 houses per community

A total of 170 triatomine bugs were collected from community houses (Fig. 5) and analyzed for infection with *T. cruzi*. We found that 62.9% of collected bugs (*n* = 107) were infected with *T. cruzi*, and that 100% of the collection sites (*n* = 8) yielded > 1 infected specimens. Geographical origin and infection rates are summarized in Fig. 6. The percentage of infected insects per locality was variable (median 63.6%, range 6.7–80.8%). The variability is likely to the small sample size in some localities. Most of the collected insects were from Isoso district, where we found *T. cruzi* infection rates of 69.3 ± 11.5% (*n* = 118 insects). In the Charagua district, 66.0 ± 20.1% (*n* = 28 insects) of the collected triatomine vectors were infected. The real-time PCR analysis showed a lower *T. cruzi* infection of the insects collected in the communities located in the interandean valleys (8.3%, *n* = 24 insects) in comparison with the two former districts located in the Chaco region.

## Discussion

The data from this field study suggest that the majority of the interviewed informants had heard about CD and had knowledge about its vectors. *Vinchucas* (*T. infestans*) were associated with CD in 70.2% cases (*n* = 480). Our data show that the majority of the affected indigenous Chiquitano (87.5%, *n* = 120), Guaraní (83.9%, *n* = 87) and Quechua (72.2%, *n* = 72) population recognized the causal relationship between the insect’s bites and CD, thus more frequently than the informants who lived in the cities Santa Cruz (67.7%, *n* = 62) and La Paz (46,1%, *n* = 89). This is explained by the fact that in the La Paz region vectors are lacking. Intriguingly, in comparison to other ethnic groups, a significantly lower percentage (48.0%) of Ayoreo informants associated triatomine bugs with CD, despite their overall good knowledge of *Triatoma* ecology. Multivariate logistic regression analyses indicate that age and educational level were significantly correlated (*P* < 0.05) with a better knowledge on the association between triatomine bugs and CD among urban informants, while there were unexpected gender differences (*P* < 0.05) among Ayoreo and Chiquitano informants concerning this knowledge. In the cities of La Paz and Santa Cruz, the knowledge about triatomine vectors and CD was mostly acquired through formal education and it linearly increased with age and educational level. In contrast, the responses of rural indigenous informants were mostly homogeneous (Fig. 3). Our data show that informants in rural areas know about triatomine bugs and CD at an early age because age had no influence (*P* > 0.05) on the association between the two. This finding is not surprising as in indigenous societies adult-level knowledge is acquired at the age of 12 to 14 and there is generally a high consensus within a given community [74, 75]. However, gender played a role among Ayoreo and Chiquitano informants. This is probably because males in these communities had higher literacy, or a better understanding of Spanish, especially in the case of Ayoreo informants. Unfortunately, Ayoreo women still lack education about CD and only 29.2% associated triatomine bugs with infection. This reveals that health education for both genders is necessary. Moreover, some Ayoreo informants reported that fumigation and health education campaigns did never take place in their villages. Clearly, in the Ayoreo ethnomedical system, disease concepts and etiologies strongly differ from Western biomedical concepts [76]. In the past, prolonged illness was generally considered of supernatural origin. The concept of curing diseases was embedded in ritual forms, shamanism and performative practices that constituted an Ayoreo “tradition” which has nowadays been entirely abandoned [77]. People no longer tell *adode* myths nor do they heal one another by sucking out or blowing away sickness with *sarode* and *ujñarone* curing chants, nor do they smoke *sidi* tobacco and *canirojnai* roots to enter into shamanic trances for spiritual healing [77]. After having been sedentarized in the second half of the twentieth century, the Ayoreo got exposed to new diseases like respiratory infections, diabetes and more recently also AIDS. There is the believe, especially among the older Ayoreo, that diseases come from the *cojñone* (non Ayoreo people) who are able to transmit them intentionally. For instance, we recorded an association between diabetes and being a community or political leader. Some Ayoreo say that diabetes in political leaders is caused by witchcraft from jealous people or that it is a punishment because of lying. Ethnomedical concepts have been largely unintelligible to anthropologists because of restricting Ayoreo acculturation to an impoverished notion of “tradition” which now represents both, a lack of shamanistic practice due to Christianization and a lack of knowledge about Western medicine.

All indigenous communities show a relatively good biological and ecological knowledge of the triatomine vectors *T. infestans* and *T. sordida* (e.g. reproduction, occurrence and general nocturnal blood sucking behavior), which is consistent with previous findings [32, 37, 41, 43]. However, the overall higher percentage regarding the association of *vinchucas* and CD significantly differed from previously conducted studies in similar endemic areas of Bolivia (Quechua [41] and Guaraní communities [43]), where there was limited knowledge of CD. This discrepancy may be attributed to the efforts of the national anti-CD campaigns in the surveyed rural areas. Indeed, a recent study within the Valleys and Chaco region identified that people recognized that CD is transmitted by *Triatoma infestans* to a similar extent as in our findings [42]. In another recent study in a rural, non-indigenous area of Santa Cruz Department all informants understood CD was transmitted by *vinchucas* and the majority recognized the symptoms of the disease [78]. However, our study is the first to provide information about the knowledge and perception concerning CD among Chiquitano and Ayoreo in Bolivia. In accordance to previous findings in endemic regions, the affected rural families have developed over time an attitude of naturalization of the problem and acceptance of triatomine bugs as they basically coexist with them [27, 29, 33, 37, 41, 42, 79]. However, contrary to other studies [39, 45], none of our informants mentioned having experienced discrimination or stigmatization because of having CD.

We found that in rural areas informants received health education about CD from non-indigenous institutions (mainly from municipal vector control personnel or health care workers). However, external transmitters had little impact on the perception regarding the threat of infection by *vincuchas.* The message of the anti-CD campaigns is therefore not convincing enough and does not transmit the nature of the risk to local communities. Knowledge and awareness of CD did not efficiently translate into changes in practice among the indigenous communities. No personal protection measures were implemented in the households visited. Preventive practices, such as restoration, amelioration and rigorous cleaning of the houses were rarely observed in the surveyed rural indigenous communities. Surprisingly, the informants did not report active searching for insects inside the dwellings or at the corrals. Moreover, a lack of hygiene and the presence of animal farming in close proximity were noticed in many indigenous communities. Domestic animals slept in the same room as family members, despite of people’s awareness that *vinchucas* attack these animals as well. Such practices are causative for transmission of the infection as reported previously [80–82]. Occasional spraying with household insecticides was the only control action undertaken by some Chiquitano informants. Most of the affected population relies on the yearly national governmental insecticide campaigns. The Bolivian national Chagas campaign puts emphasis on organized vector control but not on personal protection measures. To date, no participatory approaches have been integrated in the surveyed communities.

The absence of actions against vector infestation of the houses could be explained by the CD pathophysiology. CD is a chronic condition that remains largely silent and manifests itself in about 30% of infected people, i.e. most patients will remain asymptomatic lifelong. In addition, there is a relatively low probability of being infected even when being continuously bitten [83]. These aspects are important when trying to understand people’s behavior. The chance of getting a disease at some hypothetical future date does not seem to be a real problem. Something similar happens in Western countries where young adults still start smoking despite of being aware that smoking is associated with lung cancer [84]. Probably, the most disadvantaged sectors are the least likely to respond to health messages, as they are already under considerable strain coping with the exigencies of everyday life. Moreover, there is a divide between communities and health centers mainly because communication barriers exist in terms of language and lack of cultural understanding. The relationship between health personnel and indigenous people is sometimes plagued by mistrust [33]. Among doctors there is the tendency to perceive rural, indigenous peasants as inferior and often the prescription of medicines goes without explaining the diagnosis [33, 79, 85, 86]. For instance, we encountered Quechua and Ayoreo informants who were diagnosed with CD but did not know anything about this illness as nobody explained them what it meant “to be positive”. In few extreme cases, Quechua and Ayoreo informants reported that they were tested but never obtained the result.

Despite the efforts to control the disease, it is still highly prevalent in some of the surveyed communities in the Chaco. Our PCR analysis reveals *T. cruzi* hyperinfection (infection rates of over 70%) in domestic triatomine bugs. The difference between infected triatomine bugs in the interandean valleys and the Chaco (Fig. 6) may be because in the former, houses are temporarily abandoned (according to the agricultural cycle of the seasons) and the villages are less densely populated. Overall, the infestation of houses in this region was lower (a lower number of bugs was collected). Vector control was reported to be effective in a similar region in the inter-Andean valleys, where infestation of about 3% of dwellings was detected [87]. In both geographical regions, previous studies reported varying infection rates in *T. infestans* [16, 56, 62, 63], which ranged between 5–70%, depending on the localities and habitat of the vectors (domestic or sylvatic). Clearly, the high *T. cruzi* prevalence in domestic triatomine vectors in the Chaco is contradictory to people’s perception of *vinchucas*. Triatomine bugs were not attributed any particular (e.g. religious, ecological, ethnomedical) characteristics and no negative traditional cultural believes regarding these bugs could be recorded. Thus, it is likely that the millennial co-existence of parasite and host in this area did not induce a selection pressure to justify actions against the vectors. Indeed, the mortality of children due to *T. cruzi* infections among these communities is relatively low and many children are born from mothers already infected [88] and live long enough to get married and have children.

There is an urgent need to improve the efficacy of health education interventions. Our findings have identified some areas where campaigns or interventions could be targeted to enhance community health behavior. Educators should be better qualified. If possible, they should speak local languages and have training about ways to increase cultural sensitivity within their practices. Furthermore, innovative methods of educating residents of the local communities should be applied. Current health education models have the simple goal to impart knowledge through organized verbal instruction. However, learning always takes place in a specific socio-cultural setting. Learning in traditional rural societies is informal and experimental, taking place in an unstructured environment. Knowledge is acquired through hands-on experience and direct observations and interactions [75, 89–93] and learning abilities need social interactions to be effective [91]. Hence, the implementation of participatory learning activities could be an important strategy in the battle against CD. Events using audio-visual tools may be implemented at healthcare facilities. For example, popular theatre could be used as a tool for communication and dissemination of health information [94–96]. Plays can overcome literacy barriers. Further, workshops and seminars could be organized with community members, health center personnel and schoolteachers, as they are important intermediaries. Indeed, education of elementary school children has been found to be an effective strategy to promote behavior change in parents [97, 98]. It is of great significance that communities are actively involved in the identification of problems and needs, search for solutions and evaluation of applied measures. Successful experiences involving community participation in CD control have been reported across Latin America [42, 99–105]. Bottom-up approaches could be applied [106] putting emphasis on preventive or personal protection measures and environmental management. Long-term community-based surveillance strategies need to be applied along with government-sponsored insecticide spraying. For example, community members could be engaged in voluntary and active entomological surveillance as they were very efficient in capturing triatomine bugs during the fieldwork. *Triatoma* vectors could be searched, deposited in special containers and reported to community leaders or coordinators. Ideally, fumigation of infested houses should follow, which could also be accomplished by community members who received special training in insecticide spraying. Previous studies reported experiences with people who sprayed their own houses [102, 105]. The use of mosquito nets, window insect screens, cleaning activities, personal hygiene, mud coating of walls, and insect traps are feasible preventive measures that could be imposed by the community. The use of physical barriers (insect screens) was identified as a good alternative strategy for the control of sylvatic and peridomiciliated vectors [107–109]. Another alternative might be the use of insecticide-impregnated materials, which has been proved to be a cost-effective option [110, 111]. Also low-cost improvements using local materials have been reported as a successful intervention [112, 113].

## Conclusions

This study reveals a significant discrepancy between local knowledge of CD and the practices to reduce risk of transmission among the studied ethnic groups Ayoreo, Guaraní and Quechua. In parts, this can be explained by an insufficient understanding of vector disease transmission, but also the lack of an acute effect on health upon infection may account for the apparent indifference towards vector bites. Indigenous ethnomedical knowledge systems differ from Western concepts of chronic diseases. This was particularly evident among the Ayoreo, who also suffered from a range of other diseases, including, acute respiratory infections, acute diarrheal diseases, diabetes and AIDS [55]. Clearly, the indigenous communities in this study did not associate danger with CD as it may not be lethal among the majority of the infected people [33, 43]. Although past insecticide spraying campaigns in the Isoso region temporarily reduced the vector infestation in the villages, during this fieldwork people literally cohabited with *T. infestans*. The bugs collected in domestic dwellings among the Guaraní showed an approximately 70% overall infection, clearly indicating hyperendemic disease transmission among indigenous communities in this area. Intriguingly, no action was taken to eliminate the bugs from the dwellings. Public health programmes aim to replace “false” beliefs with “accurate” knowledge. By changing community knowledge, it is assumed, behavior will also be changed [44]. Although education programmes have raised awareness, they did not greatly affect the behavior of people. Accordingly, a significantly high proportion of the affected indigenous informants had some knowledge of the disease vector but they did not translate it to prevention or protection practices. Therefore, a change of perception regarding triatomine bugs as being potentially lethal is indispensable. While it is unnecessary that the affected population understands CD from a biomedical perspective, the scientific information needs to be translated into culturally appropriate categories that are sensitive to indigenous values, traditions and motivations [79]. An early and thorough education of children on the danger of triatomine bugs should accompany the current anti-CD campaigns. Continued vector control activities (e.g. regular collecting of bugs from dwellings) with community-based health intervention programmes using strategies of community participation could be an important strategy to break the vicious circle between hyperendemic CD communities and hyperinfected vectors infesting their houses. Two decades after disease intervention, the rural populations still suffer from *T. cruzi* infections, which could be reduced solely by changing the widespread view on the nature of threat by vector bites. Overall, we believe that through the involvement and experience of local people the impact of current interventions should be translated into changes of practice.

## Abbreviations

AIDS: Acquired Immune Deficiency Syndrome
BID: Banco Interamericano de Desarrollo
CD: Chagas disease
PAHO: Pan American Health Organization
PCR: polymerase chain reaction
WHO: World Health Organization
